# Outcomes of frozen embryo transfers from a large monocentric cohort (2982 cycles): towards a preferential use of cycles with a *corpus luteum* for endometrial preparation

**DOI:** 10.3389/fendo.2026.1808602

**Published:** 2026-05-05

**Authors:** Julie Labrosse, Emilie Haab, Frida Entezami, Nathalie Massin

**Affiliations:** 1 Department of Reproductive Medicine, American Hospital of Paris, Neuilly-sur-Seine, France; 2 Reproductive Medicine Department, Intercommunal Hospital of Créteil, Créteil, France

**Keywords:** artificial cycle, frozen embryo transfer, hormone replacement therapy, live birth, natural cycle

## Abstract

**Introduction:**

To determine if endometrial preparation protocols for frozen embryo transfers (FET) impact clinical and neonatal outcomes.

**Methods:**

A monocentric cohort study including all first autologous FET cycles of a single blastocyst from January 1st, 2020 to September 30th, 2023. The primary endpoint was live birth (LBR). Secondary endpoints included hCG-positive pregnancy (> 100 IU/l), ongoing pregnancy (OPR), miscarriage, birthweight and term. Outcomes were also analyzed by subgroup: Day 5/Day 6 embryos. Among the 2982 FET cycles, 1507 (50.5%) were artificial cycles (AC) and 1475 (49.5%) were cycles with a *corpus luteum* (CLC). Univariate and multivariate analyses were performed using logistic regression models adjusting on age at transfer, age at oocyte retrieval, body mass index (BMI), indication and duration of infertility, IVF/ICSI, AMH, embryo quality, ovulatory status and year of transfer.

**Results:**

Age at the time of transfer (35.3 *vs.* 36.0 years old) and age at oocyte retrieval (34.8 *vs.* 35.4 years old) were significantly lower in AC compared to CLC. After adjustment, CLC significantly increased LBR (aOR = 1.39 [1.12;1.73], *P* = 0.003) and OPR (aOR = 1.39 [1.12;1.74], *P* = 0.003) and significantly decreased miscarriage (aOR=0.63 [0.49;0.79], *P* = 0.0001) compared to AC. In subgroup analyses, Day 5 embryos had significantly higher LBR, OPR, hCG-positive rates and lower miscarriage rates compared to Day 6 embryos. Term of birth did not differ between the two groups.

**Conclusion:**

Our study pleads for a preferential use of CLC protocols for endometrial preparation for FET in ovulatory women.

## Introduction

The number of frozen embryo transfers (FET) is increasing worldwide and is a key component of success in assisted reproductive outcomes (1). Two types of protocols are used for endometrial preparation in FET. On one hand, artificial cycles (AC) consist in the sequential administration of estrogen and progesterone. On the other hand, cycles with an ovulation and a subsequent *corpus luteum* (CLC) can be used, including natural cycles, modified natural cycles, and stimulated cycles.

Whereas ovulatory cycles lead to the development of a CL, AC induce endometrial growth and transformation without a CL. There is growing evidence that the absence of a CL in AC leads to adverse obstetrical and neonatal outcomes (2–8). Studies have reported higher risks of early pregnancy loss, gestational diabetes, vascular disorders such as gestational hypertension or pre-eclampsia, placental pathology, postpartum hemorrhage, and higher birthweights with AC compared to CLC (2–8). The supraphysiological concentrations of estrogen and progesterone in AC may increase the risk of vascular disorders (9). The absence of a CL may also be deleterious because the CL does not only produce estrogen and progesterone but also vasoactive products such as relaxin, vascular endothelial growth factor (VEGF) and angiogenic metabolites of estrogen that play a role in placentation and early pregnancy development (10).

AC remain widely used worldwide as it is more convenient in terms of organization for centers by providing more control over timing of FET. Studies have evaluated the effects of these two types of endometrial preparation protocols on pregnancy outcomes in FET but their findings remain debatable (11–15). Despite the convenience of using AC protocols to plan FET, the higher incidence of obstetrical and neonatal adverse outcomes associated to AC should be considered. The objective of our study is to compare the clinical outcomes between CLC and AC for endometrial preparation in FET to determine which protocol should be used while minimizing the risk of obstetrical and neonatal complications.

## Methods

### Study participants

**Figure 1:**
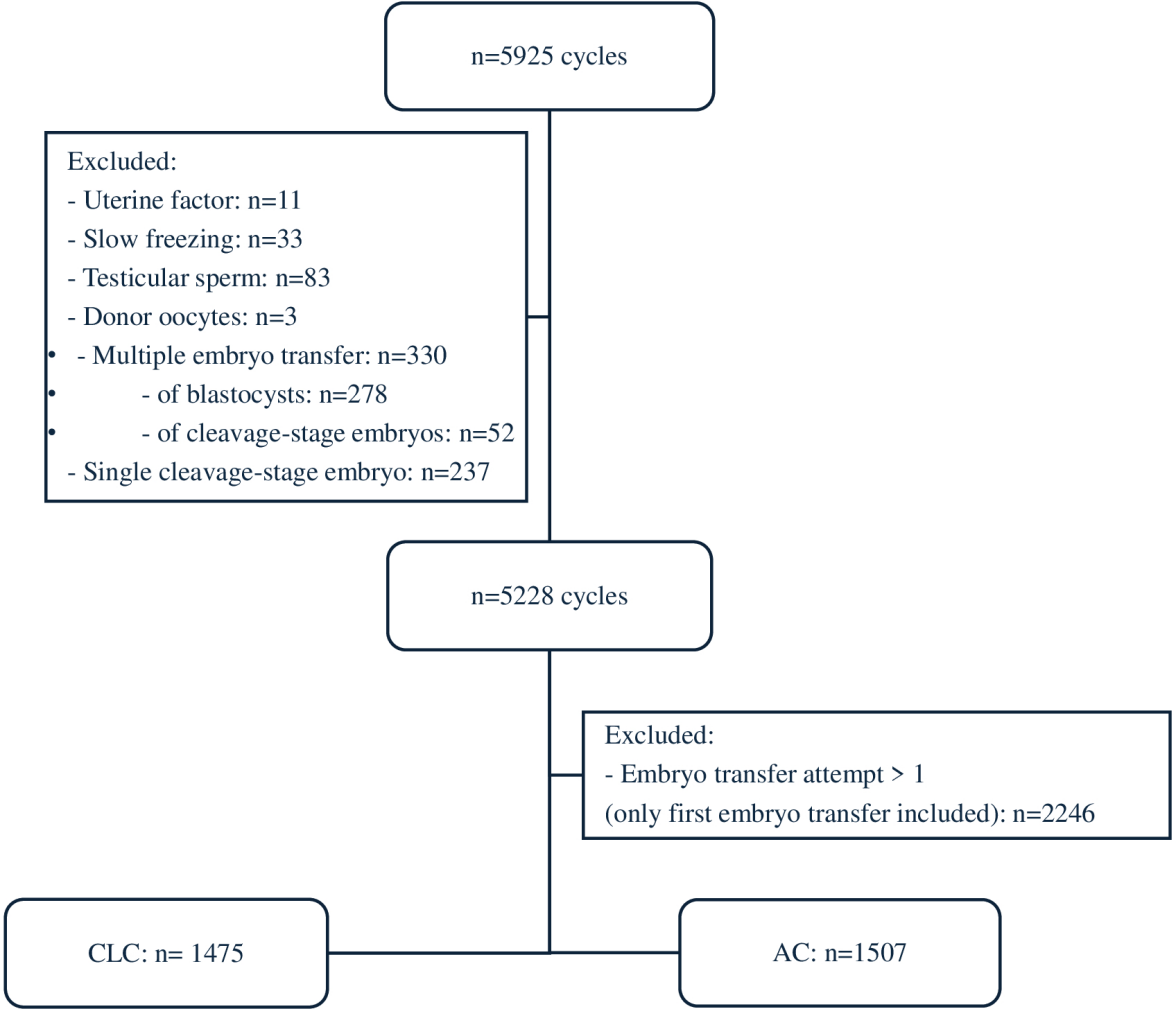
Flow diagram.

This is a retrospective monocentric cohort study including all FET cycles from January 1st, 2020 to September 30th, 2023 performed at the American Hospital of Paris, France. Every FET of a single vitrified blastocyst was included. Only results of the first FET were analyzed to avoid the potential bias of patients who previously had unsuccessful FET. This study only included untested embryos, as preimplantation genetic testing for aneuploidy (PGT-a) is not allowed in France. We excluded cases of uterine infertility, embryos obtained after slow freezing, embryos originating from fertilization with testicular sperm or from egg donation, FET of more than one embryo, and cleavage stage embryos (**Figure 1**). Our laboratory does not transfer Day 7 embryos. For each patient, baseline characteristics were retrieved from electronic patient files registered in our database.

### Endometrial preparation protocols

The choice of endometrial preparation protocol was based on patient characteristics and history. FET are performed every day in our center, including on Saturdays and Sundays. The AC group included all protocols consisting of the exogenous administration of estrogen and progesterone, *i.e.* without a CL (natural oral estradiol 6mg/day and vaginal progesterone 800mg/day +/- additional subcutaneous progesterone if necessary, all continued until 10 weeks of gestation). The CLC group included all ovulatory protocols (*i.e.* with a CL), with or without trigger. All CLC had luteal phase support (vaginal progesterone 800mg/day). In all cases, one single blastocyst was transferred (16–20).

### Data analysis and statistics

After descriptive analyses, baseline characteristics were compared between the AC and CLC groups. Continuous variables were expressed as mean ± standard deviation (SD) or median [interquartile range] as appropriate and compared using Student’s t-test or the Mann–Whitney U test. Categorical variables were expressed as counts (percentages) and compared using the chi-square test or Fisher’s exact test, as appropriate.

The primary outcome was live birth rate (LBR). Secondary outcomes included hCG-positive pregnancy (>100 IU/L), ongoing pregnancy (OPR), miscarriage, birthweight, and term of birth. Additionally, we performed an adjusted analysis of birthweight including maternal age, body mass index (BMI), anti-Mullerian hormone (AMH), gestational age at delivery, and sex of the newborn. Miscarriage was defined as pregnancy loss occurring after a positive human chorionic gonadotropin (hCG) test (>100 IU/L) and before the end of the first trimester. Miscarriage rates were calculated among hCG-positive pregnancies.

Associations between endometrial preparation protocol (CLC *vs.* AC) and binary outcomes were evaluated using logistic regression models. Multivariable models were adjusted for potential confounders identified *a priori* based on clinical relevance, including age at oocyte retrieval, BMI, AMH levels, ovulatory status, type and duration of infertility, fertilization method (*in vitro* fertilization (IVF) *vs.* intracytoplasmic sperm injection (ICSI)), embryo quality, and embryo stage (Day 5 *vs.* Day 6). Odds ratios (ORs) with 95% confidence intervals (CIs) were reported. As only the first FET cycle per patient was included, no clustering effect was expected.

Interaction terms were not included in the models, as the study was not specifically powered to assess interaction effects.

To avoid overfitting and reduce bias associated with data-driven selection procedures, all clinically relevant covariates were retained in the final models without stepwise selection. Multicollinearity between covariates was assessed using variance inflation factors.

Continuous outcomes, including birthweight, were analyzed using linear regression models. Model assumptions, including normality of residuals, were assessed graphically. Results are presented as mean differences with 95% CIs.

Embryo stage (Day 5 *vs.* Day 6) was included as a covariate in the multivariable models.

Missing data were handled using a complete-case analysis, as the proportion of missing values was low and did not suggest a systematic pattern.

All statistical tests were two-sided, and a *P*-value <0.05 was considered statistically significant. Analyses were performed using R statistical software (R Foundation for Statistical Computing, Vienna, Austria).”

### Ethical approval

This study was led according to institutional and ethical rules regarding research on patients and specimens. According to the French regulation, no written consent is required for retrospective analyses. The study was approved by the institutional ethical committee board.

## Results

A total of 2982 FET cycles were included. Among the 2982 FET cycles, 1507 (50.5%) were AC and 1475 (49.5%) were CLC protocols. Among the CLC protocols, 848 cycles (57.5%) used hCG trigger.

### Baseline characteristics

Cycle characteristics are detailed in **Table 1**. In AC, patients were significantly younger at the time of transfer (mean age: 35.4 *vs.* 36.0 years old, respectively) and at retrieval (mean age: 34.9 *vs.* 35.4 years old, respectively). They also had significantly higher BMI and AMH levels. Ovulation status was also significantly different between AC and CLC patients.

**Table 1:** Baseline characteristics.

Variable	CLC (n=1475)	AC (n=1507)	*P*
Age at transfer (mean (SD))*	36.0 (4.2)	35.4 (4.5)	< 0.001
Age category*			0.07
< 35 years old	696 (47.2)	772 (51.2)	
35–39 years old	436 (29.6)	423 (28.1)	
> 39 years old	343 (23.3)	312 (20.7)	
Age at oocyte retrieval (mean (SD))*	35.4 (4.2)	34.9 (4.5)	0.002
BMI (mean (SD))*	22.88 (4.0)	23.31 (4.5)	0.02
Duration of infertility (mean (SD))*	47.34 (28.6)	46.38 (28.7)	0.37
Secondary infertility (%)*	986 (66.8)	1044 (69.3)	0.05
Ovulation (%)*			< 0.001
WHOI (hypogonadotropic hypogonadism)	6 (0.5)	22 (1.6)	
WHOIIa (normogonadotropic normogonadic ovarian dysfunction non-PCOS)	151 (11.6)	135 (10.0)	
WHOIIb (normogonadotropic normogonadic ovarian dysfunction PCOS)	146 (11.2)	282 (20.8)	
WHOIII (hypergonadotropic hypogonadism)	209 (16.1)	126 (9.3)	
No ovulation disorder (%)	790 (60.7)	791 (58.3)	
Active smoking (mean (SD))	0.11 (0.31)	0.10 (0.30)	0.24
Endometriosis (%)	144 (9.8)	169 (11.2)	0.20
Endometrial thickness (mean (SD))	8.89 (1.9)	8.90 (1.7)	0.89
AMH (mean (SD))*	2.92 (2.6)	3.90 (3.7)	< 0.001
IVF/ICSI (%)*			0.73
IVF	631 (43.0)	663 (44.1)	
ICSI	800 (54.5)	806 (53.6)	
Embryo quality (%)*			0.29
Top	538 (39.0)	586 (41.4)	
Good	763 (55.4)	745 (52.6)	
Average	75 (5.4)	79 (5.6)	
Low	2 (0.1)	6 (0.4)	
hCG > 100 IU (%)	614 (41.8)	621 (41.3)	0.80

*variable included in multivariable analyses.

### LBR

**Figure 2:**
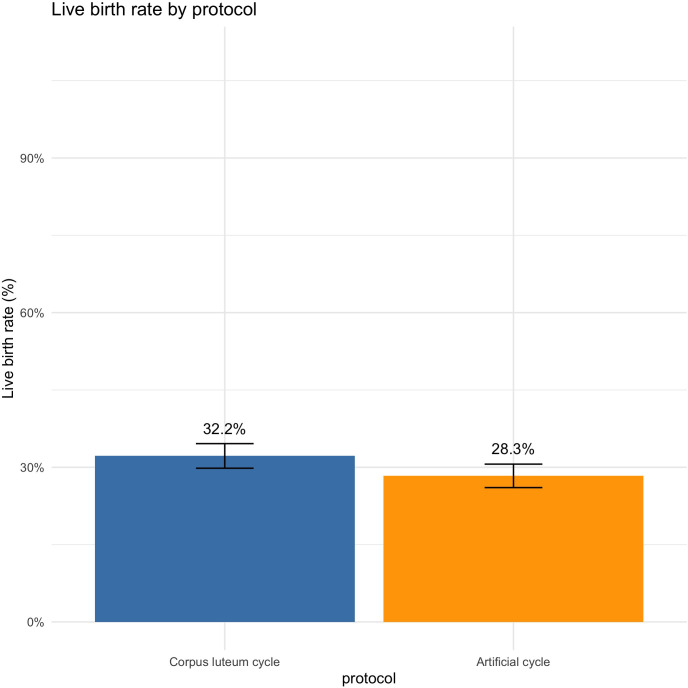
Live birth rate by protocol.

In univariate analysis, there were 32.2% of LBR in the CLC group *vs.* 28.3% in the AC group (OR = 1.20 [1.03;1.41], *P* = 0.02). After adjustment on age at transfer, age at oocyte retrieval, BMI, AMH, ovulation status, type and duration of infertility, IVF/ICSI and embryo quality, CLC significantly increased LBR compared to AC (aOR=1.39 [1.12;1.73], *P* = 0.003) (**Figure 2**). When restricted to strictly ovulatory women (790 in CLC and 791 in AC, *i.e.* 1581 total), LBR were significantly higher in CLC *vs.* AC (33.2% vs. 26.9%, (OR = 1.35 [1.08;1.69], *P* = 0.007) in univariate analysis. After adjustment, LBR remained significantly higher in CLC vs. AC (aOR=1.39 [1.02;1.89], *P* = 0.037).

### Secondary endpoints

**Figure 3:**
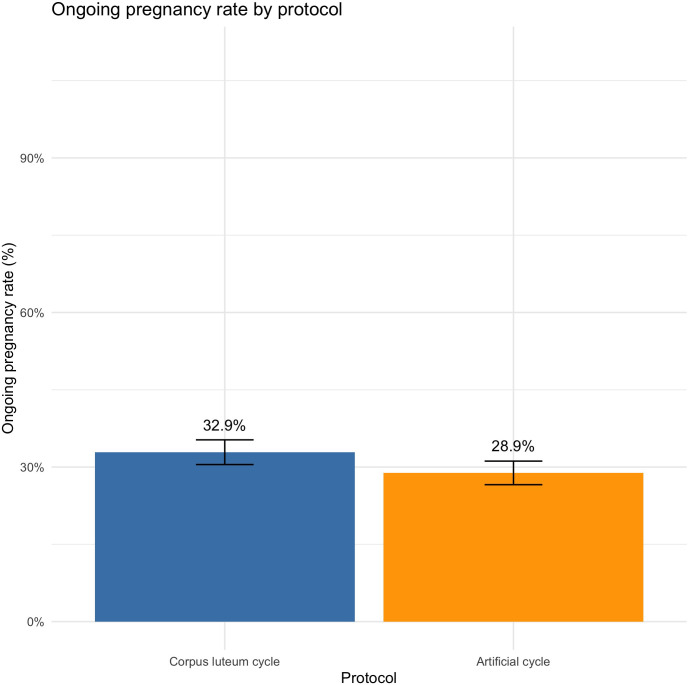
Ongoing pregnancy rate by protocol.

In univariate analysis, there were 32.9% of OPR in the CLC group *vs.* 28.9% in the AC group (OR = 1.21 [1.03;1.41], *P* = 0.02). After adjustment on age at transfer, age at oocyte retrieval, BMI, AMH, ovulation status, type and duration of infertility, IVF/ICSI and embryo quality, OPR were significantly higher in CLC compared to AC (aOR = 1.40 [1.12;1.74], *P* = 0.003) (**Figure 3**). No difference was found for hCG-positive pregnancies between CLC and AC cycles.

**Figure 4:**
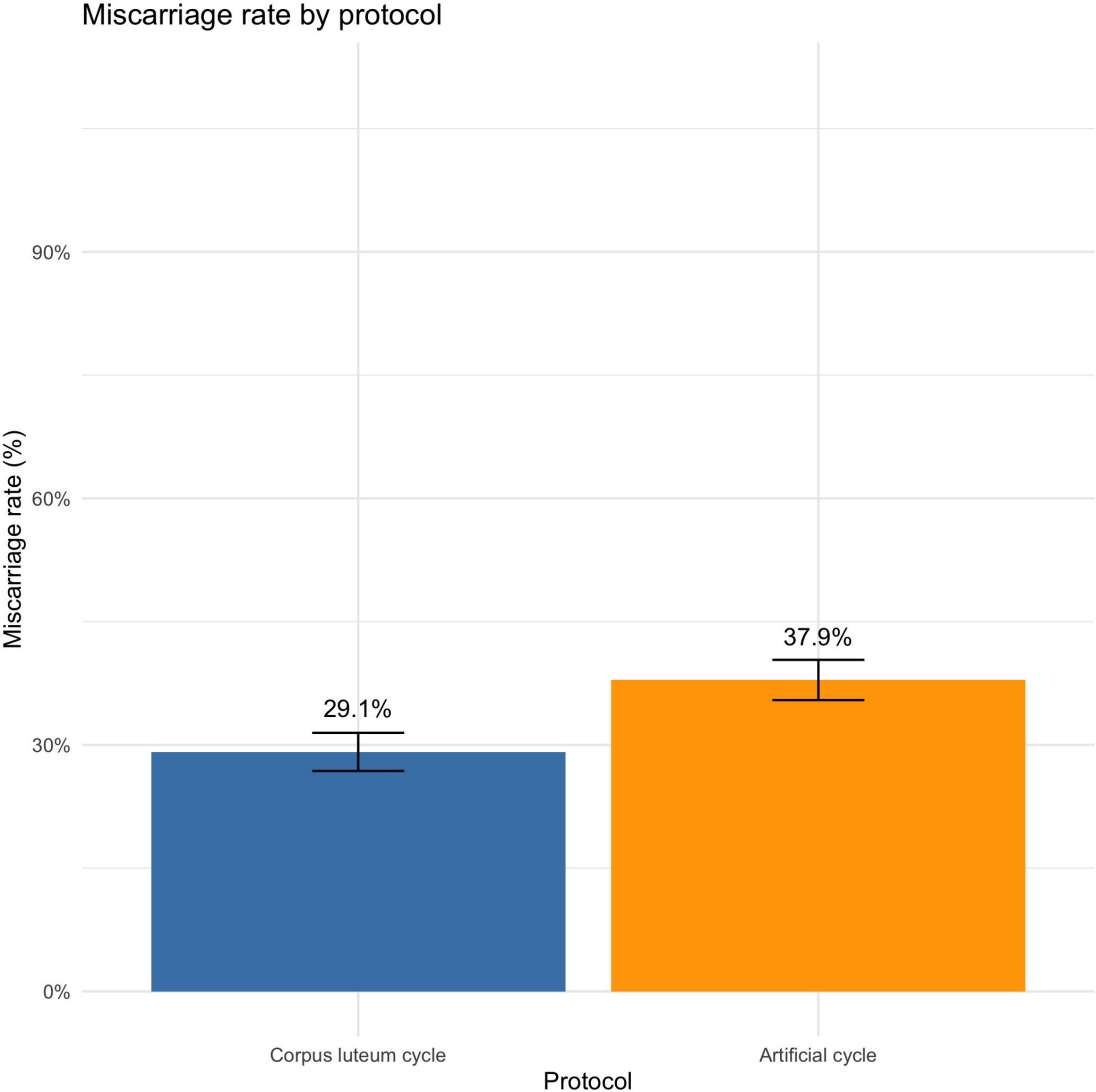
Miscarriage rate by protocol.

In univariate analysis, there were 29.1% miscarriages in the CLC group *vs.* 37.9% in the AC group (OR = 0.67 [0.54;0.84], *P* = 0.001). After adjustment, miscarriage was significantly lower in CLC *vs.* AC (aOR = 0.63 [0.49;0.79], *P* = 0.0001) (**Figure 4**). Age at retrieval was significantly associated to miscarriage. We found that miscarriage increase of 6% per additional year of age. In the analysis by age category (< 35 years old, 35–39 years old, > 39 years old), miscarriage was significantly lower in CLC for women < 35 years old (OR = 0.64 [0.47; 0.88], *P* = 0.006) and women aged between 35–39 years old (OR = 0.51 [0.33; 1.79], *P* = 0.002). However, miscarriage was not impacted by the type of endometrial preparation protocol for women aged > 39 years old (OR = 0.93 [0.53; 1.46], *P* = 0.81) (**Table 2**).

**Table 2:** Miscarriage by age group.

Age	CLC	AC	*P*
< 35 years old	26.2	33.4	0.006
35–39 years old	25.6	41.0	0.002
> 39 years old	46.3	51.0	0.81

Only Day 5 embryos (n = 2481) and Day 6 embryos (n=500) were included in our study. Day 5 embryos were significantly associated to higher LBR (32.4% *vs.* 19.4%, *P* < 0.001), OPR (33.1% *vs.* 19.8%, *P* < 0.001), hCG-positive pregnancy (44.2% *vs.* 28.0%, *P* < 0.001) and lower miscarriage (32.2% *vs.* 43.6%, *P* = 0.003) compared to Day 6 embryos. Although formal stratified analyses according to embryo stage were not performed, the association between endometrial preparation protocol and outcomes appeared consistent with the overall results.

Term of birth did not differ between the two groups.

AC was associated to significantly higher birthweights (+41.4g) compared to CLC. In an additional analysis adjusted for maternal age, BMI, AMH, gestational age at delivery and sex of the newborn, gestational age and sex were significantly associated with birthweight, as expected. However, the type of endometrial preparation protocol was not significantly associated with birthweight after adjustment. The rate of small for gestational age (SGA: < 10^th^ percentile) and large for gestational age (LGA > 90^th^ percentile) were not significantly different between the two protocols of endometrial preparation (for SGA: 3.8% in AC *vs.* 6.6% in CLC (*P* = 0.10) for LGA: 25.6% in AC *vs.* 22.6% in CLC (*P* = 0.34) (**Table 3**).

**Table 3:** Obstetrical and neonatal outcomes.

Variable	CLC (n=332)	AC (n=317)
Birthweight (mean (SD))	3233 (510)	3329 (494)
Gestational age (mean (SD))	39.4 (1.9)	39.6 (1.7)
SGA (n)	22 (6.6%)	12 (3.8%)
LGA (n)	75 (22.6%)	81 (25.6%)

## Discussion

After adjustment on significant variables (age at transfer, age at oocyte retrieval, BMI, AMH, ovulation status, type and duration of infertility, IVF/ICSI and embryo quality), LBR and OPR were significantly higher with CLC compared to AC. Interestingly, results were comparable between CLC and AC for hCG-positive pregnancies, but miscarriage was significantly higher with AC compared to CLC. This underlines that the endometrial preparation protocol does not affect implantation in itself, but the following steps. Only results of the first FET were analyzed to avoid the potential bias of patients who previously had unsuccessful FET. Compared to CLC, patients in the AC group were significantly younger, had a significantly higher BMI and AMH. Ovulatory status was also different between AC and CLC, since patients with ovulation disorders due to polycystic ovarian syndrome (PCOS) were more likely to receive endometrial preparation by AC. Consistently, AMH levels were significantly higher in the AC group *vs.* CLC. PCOS is also known to be a risk factor for metabolic disorders and weight gain. Coherently, BMI was slightly higher in the AC group *vs.* CLC, although it is important to note that BMI in the two groups remain in the same BMI category. The fact that better clinical outcomes were observed in CLC *vs.* AC despite patients being significantly younger at the time of transfer and at retrieval in AC further pleads for a preferential use of CLC for endometrial preparation. Furthermore, sensitivity analyses restricted to strictly ovulatory patients confirm our results.

In line with our findings, a previous study of 4470 FET cycles showed higher rates of preclinical and clinical pregnancy losses in AC protocols (21). A large multicenter cohort study of 14–421 FET cycles (AC: n = 8139; CLC: n = 6282) found that the early pregnancy loss rate was significantly higher in AC compared to stimulated cycles (OR 1.63 [1.35-1.97]; *P* < 0.0001) and to natural cycles (OR 1.87 [1.55-2.26]; *P* < 0.0001) (6). Consistently, a systematic review and meta-analysis of thirty studies comparing obstetrical and neonatal outcomes according to the type of endometrial preparation protocol reported that CLC was associated to a significantly lower risk of early pregnancy loss compared to AC (OR [0.73; 0.61–0.86] (22). Interestingly, in our analysis by age category, miscarriage was significantly higher with AC for women aged < 35 years old and those aged between 35–39 years old, but not for women aged > 39 years old. Knowing that the CL in ovulatory cycles is responsible of the production of estrogen, progesterone and vasoactive substances which are hypothesized to be important for initial placentation and pregnancy development, these findings might imply that the CL is less efficient in older women (10). Some bovine models for the study of reproductive aging in women have suggested that both functional and structural decline of CL occurs with aging (23). The aneuploidy rate is known to increase with age and reaches 65% and above for women aged > 40 years old, according to studies (24, 25). Hence, in our category of patients aged > 39 years old, the impact of aneuploidy on miscarriage might surpass the impact of the endometrial preparation.

Despite an increasing number of FET performed worldwide, the optimal method to prepare the endometrium for FET continues to be debated. The main aim of endometrial preparation is to synchronize ET with endometrial receptivity. In line with previous systematic reviews and previous meta-analyses (15, 26), a randomized controlled trial (RCT) of 145 FET (n = 72 in CLC; n = 73 in AC) did not find any difference in pregnancy outcomes between the two groups in terms of LBR and implantation rates (11). However, the fact that the sufficient number of patients needed in each arm was not reached, the fact that both cleavage and blastocyst stage embryos were included and the absence of luteal phase support in the CLC group might impact these results. Similarly, a randomized controlled, non-inferiority trial of CLC *vs.* AC for FET (n = 495 in CLC; n = 464 in AC) showed no difference between the two protocols in terms of LBR and OPR (12). However, the study suffered from a higher rate of dropout than expected explaining the fact that the required sample size was not reached, that both cleavage and blastocysts were included and that results were not restricted to the first embryo transfer. Given these debated data so far, significantly increased LBR in CLC observed in our study suggests a larger use of CLC to optimize outcomes.

In addition to the clinical outcomes of FET, literature has also suggested that AC was associated to more obstetrical and neonatal complications. Among these, AC has been described as increasing hypertensive disorders of pregnancy (3, 7, 22, 27, 28). In the systematic review and meta-analysis of thirty studies by Zaat *et. al*, AC was significantly associated to increased hypertensive disorders of pregnancy, pre-eclampsia, placenta previa and postpartum hemorrhage. An important retrospective cohort study from the Japanese registry found increased risks of hypertensive pregnancy disorders and placenta accreta in comparison to pregnancies after CLC (3). These increased risks might be explained by the absence of a CL (4, 29), with a potential role of relaxin, a potent vasodilatator, that is produced solely by the CL during human pregnancy. In this study, we describe that the mean birthweight was lower with CLC in univariate analysis, which has also been reported in the meta-analysis by Zaat et al. (mean difference 26.4 g), in the meta-analysis of Rosalik et al. (mean difference 47.4 g) (30), and in a big retrospective analysis of 9267 cycles with live-born singletons (n= 6523 for NC, n= 2744 for NC) (31). This is particularly interesting knowing that higher birthweights were previously reported in FET *vs.* fresh embryo transfer and spontaneous pregnancy (32). However, the type of endometrial preparation protocol was not significantly associated to birthweight after adjustment, and our study is limited by the fact that other obstetrical and neonatal complications such as hypertensive disorders, gestational diabetes, placental complications, or neonatal intensive care unit admissions are not reported.

In all, AC remain widely used worldwide, as it is more convenient in terms of organization for centers by providing more control over timing of FET. Results of our study pleads for a preferential use of CLC for endometrial preparation for ovulatory patients, leading to better clinical outcomes. To facilitate a wider use of CLC, new protocols such as the natural proliferative phase strategy may be an effective and safe alternative to both NC and AC (33, 34). Further investigation is warranted, with prospective studies and obstetrical and neonatal data. For future research, finding strategies to facilitate the practicality of CLC is crucial to expand their use.

## Data Availability

The raw data supporting the conclusions of this article will be made available by the authors, without undue reservation.
